# Cardiometabolic Risk Factors and Cognitive Impairment Among Community-Dwelling Older Adults in Rural India: A Cross- Sectional Analysis of the Longitudinal Ageing Study in India (LASI) Wave 1

**DOI:** 10.21203/rs.3.rs-9652375/v1

**Published:** 2026-06-01

**Authors:** A. Arul Murugan, S. Shobana, K. Vanaja, J. Sathya

**Affiliations:** Gandhigram Institute of Rural Health and Family Welfare Trust (GIRHFWT); Kasturba Hospital; Gandhigram Institute of Rural Health and Family Welfare Trust (GIRHFWT); Gandhigram Institute of Rural Health and Family Welfare Trust (GIRHFWT)

**Keywords:** Cognitive impairment, Cardiometabolic risk, Rural ageing, Older adults, India, LASI, Dementia prevention, Community medicine

## Abstract

**Background:**

India is experiencing a rapid demographic transition with an ageing population increasingly burdened by cardiometabolic disease. Despite growing evidence from high-income countries linking cardiovascular risk factors with late-life cognitive decline, data from rural South Asia remain scarce. We examined the prevalence of cognitive impairment and its association with cardiometabolic and sociodemographic determinants among community-dwelling older adults in India, with a focus on rural residence.

**Methods:**

We conducted a cross-sectional analysis using Wave 1 data (2017–18) of the Longitudinal Ageing Study in India (LASI), a nationally representative survey of adults aged 45 years and above (analytic sample: 31,464 individuals aged ≥ 60 years). Cognitive impairment was defined using the LASI composite cognitive index encompassing memory, orientation, arithmetic, executive function, and object naming domains. Cardiometabolic risk factors included self-reported hypertension, diabetes mellitus, tobacco use, and physical inactivity. Covariates included age, sex, education, rural–urban residence, and depressive symptoms. Binary logistic regression was used to estimate crude and adjusted odds ratios (aOR) with 95% confidence intervals.

**Results:**

The overall prevalence of cognitive impairment was 10.8% (rural 12.8% vs. urban 6.0%; p < 0.001). In adjusted models, rural residence (aOR 1.68; 95% CI 1.52–1.87), female sex (aOR 2.18; 95% CI 1.99–2.38), no formal education (aOR 3.87; 95% CI 3.35–4.47), hypertension (aOR 1.19; 95% CI 1.10–1.29), tobacco use (aOR 1.26; 95% CI 1.16–1.37), physical inactivity (aOR 1.28; 95% CI 1.18–1.38), and depression (aOR 1.83; 95% CI 1.68–1.99) were independently associated with cognitive impairment. Diabetes mellitus did not reach statistical significance after adjustment (aOR 1.11; 95% CI 0.99–1.24; p = 0.072).

**Conclusions:**

Rural older adults in India face a substantially higher cognitive impairment burden driven by compounding cardiometabolic and sociodemographic vulnerabilities. Integrated community-based screening and cardiometabolic risk reduction strategies are urgently needed, particularly in rural settings. These findings have direct implications for the design of geriatric care programmes in primary health care settings.

## 1. INTRODUCTION

The global burden of dementia is projected to reach 152 million by 2050, with low- and middle-income countries (LMICs) expected to bear nearly two-thirds of the burden [1]. India alone, with a population of over 140 million adults aged 60 years and above, faces a rapidly escalating dementia crisis. The 2024 Lancet Commission on Dementia Prevention identified 14 modifiable risk factors—including hypertension, diabetes, obesity, physical inactivity, tobacco use, and depression—that collectively account for nearly 40% of attributable dementia risk [2]. However, the evidence underlying these risk factor profiles derives predominantly from high-income country cohorts, leaving a critical knowledge gap for the Indian context.

The Longitudinal Ageing Study in India (LASI), launched in 2017–18 as a nationally representative survey of over 72,000 adults aged 45 years and above, offers a landmark scientific resource to address this gap. LASI and its dementia substudy (LASI-DAD) represent India’s most comprehensive population-based dataset on cognitive aging, incorporating standardised cognitive assessments, biomarker data, and detailed sociodemographic and health information [3, 4].

Rural older adults in India face a distinct risk profile compared to their urban counterparts: lower educational attainment, greater exposure to solid fuel combustion, higher prevalence of anaemia and infectious disease burden, and limited access to preventive cardiometabolic care [5, 6]. Yet, despite constituting approximately 65% of India’s older adult population, rural-specific evidence on cardiometabolic determinants of cognitive decline remains sparse.

Using LASI Wave 1 data, this study aimed to: (i) estimate the prevalence of cognitive impairment among community-dwelling older adults aged 60 years and above; (ii) examine rural–urban differentials in cognitive impairment; and (iii) identify independent cardiometabolic and sociodemographic determinants of cognitive impairment, with an emphasis on modifiable risk factors amenable to primary health care intervention in rural India.

## 2. METHODS

### 2.1 Study Design and Data Source

This was a secondary analysis of cross-sectional data from Wave 1 of the Longitudinal Ageing Study in India (LASI), conducted between 2017 and 2019 by the International Institute for Population Sciences (IIPS), Mumbai, in collaboration with Harvard T.H. Chan School of Public Health and the University of Southern California, with funding from the Ministry of Health and Family Welfare, Government of India, and the National Institute on Aging (NIA), USA [3].

The LASI used a multistage stratified area probability cluster sampling design, with separate three-stage (rural) and four-stage (urban) sampling frames, achieving nationally representative coverage across 29 states and 6 union territories. Wave 1 enrolled 72,262 adults aged 45 years and above, along with their spouses irrespective of age. For the present analysis, we restricted the sample to individuals aged 60 years and above (n = 31,464) for whom complete cognitive assessment data were available.

The LASI data are publicly available upon registration at the IIPS data portal (https://www.iipsindia.ac.in/lasi). This secondary analysis was conducted under the terms of the LASI data use agreement and did not require additional ethical approval.

### 2.2 Outcome Variable

The primary outcome was cognitive impairment, defined using the LASI composite cognitive index. The cognitive assessment module of LASI, adapted from the Health and Retirement Study (HRS) cognitive battery, evaluated five domains: (i) memory (immediate and delayed word recall; maximum 10 words), (ii) orientation (day, month, year, day of the week; maximum 4), (iii) arithmetic function (serial 7 subtraction; maximum 5), (iv) executive function/attention (backward counting; maximum 2), and (v) object naming (two objects shown on cards; maximum 2). The composite score ranged from 0 to 43. Cognitive impairment was defined as a composite score below the lowest tertile of the age- and education-specific distribution, consistent with prior LASI-based studies [5, 7].

### 2.3 Exposure Variables

Primary cardiometabolic exposures were self-reported hypertension (ever diagnosed by a physician), diabetes mellitus (ever diagnosed), current tobacco use (smoking or smokeless), and physical inactivity (no moderate or vigorous physical activity in the past week). Sociodemographic covariates included: age (continuous), sex, educational attainment (categorised as no formal education, 1–4 years, 5–9 years, ≥ 10 years), rural–urban residence, and depressive symptoms (Geriatric Depression Scale [GDS] short-form, GDS ≥ 3 defined as probable depression [8]).

### 2.4 Statistical Analysis

Descriptive statistics were computed for all variables, stratified by rural–urban residence. Chi-squared tests for categorical variables and independent samples t-tests for continuous variables were used to assess bivariate associations. Binary logistic regression was performed in two steps: Model 1 (unadjusted) estimated crude odds ratios (OR) for each exposure; Model 2 (adjusted) included all covariates simultaneously to estimate adjusted ORs (aOR) with 95% confidence intervals. Survey-weighted estimates accounting for the complex sampling design were computed using the svydesign and svyglm functions in R (version 4.3.0) using the ‘survey’ package. Statistical significance level was set at p < 0.05. STROBE reporting guidelines for cross-sectional studies were followed.

## 3. RESULTS

### 3.1 Characteristics of the Study Population

The analytic sample comprised 31,464 older adults aged 60 years and above (52.0% female; mean age 68.4 ± 7.2 years). Of these, 22,108 (70.3%) resided in rural areas. Rural participants had significantly lower mean educational attainment (3.0 ± 4.6 vs. 6.8 ± 5.5 years; p < 0.001) and lower prevalence of cardiometabolic conditions including hypertension (35.1% vs. 43.7%) and diabetes (13.0% vs. 24.3%), though they had higher prevalence of tobacco use (32.9% vs. 23.2%) and physical inactivity was less common in rural areas (40.0% vs. 53.3%). Depressive symptoms were more prevalent in rural participants (29.9% vs. 23.5%; p < 0.001). Full baseline characteristics are presented in Table 1.

### 3.2 Prevalence of Cognitive Impairment

The overall prevalence of cognitive impairment was 10.8% (n = 3,385). Cognitive impairment was significantly more prevalent among rural than urban participants (12.8% vs. 6.0%; p < 0.001). Mean composite cognitive scores were markedly lower in rural areas (23.9 ± 8.0 vs. 29.1 ± 7.6; p < 0.001). Cognitive impairment was more common among women (13.4% vs. 7.8%), those with no formal education (18.2%), and those aged 80 years and above (24.7%).

### 3.3 Determinants of Cognitive Impairment: Logistic Regression

In adjusted analysis, rural residence remained independently associated with cognitive impairment (aOR 1.68; 95% CI 1.52–1.87) after controlling for all covariates. Female sex (aOR 2.18), no formal education (aOR 3.87), and depressive symptoms (aOR 1.83) were the strongest independent predictors. Among cardiometabolic factors, hypertension (aOR 1.19), tobacco use (aOR 1.26), and physical inactivity (aOR 1.28) were all independently and significantly associated with cognitive impairment. Diabetes mellitus did not achieve statistical significance in the adjusted model (aOR 1.11; 95% CI 0.99–1.24; p = 0.072). Full regression results are presented in Table 2.

## 4. DISCUSSION

This nationally representative analysis of LASI Wave 1 data reveals a substantial and rural-concentrated burden of cognitive impairment among older adults in India. One in eight rural older adults met criteria for cognitive impairment—more than double the prevalence observed in urban areas. The magnitude of the rural–urban disparity (aOR 1.68) persisted after adjustment for education, cardiometabolic risk, and depressive symptoms, indicating that structural and environmental factors unique to rural settings contribute independently to cognitive aging trajectories.

Our findings on cardiometabolic determinants are broadly consistent with the 2024 Lancet Commission framework. Hypertension, tobacco use, and physical inactivity each independently predicted cognitive impairment, underscoring the relevance of established cardiovascular risk reduction strategies for dementia prevention in India. The non-significant association with diabetes mellitus in the adjusted model is an intriguing finding. Prior LASI-DAD analyses have documented that, unlike Western populations where higher HbA1c is associated with worse cognition, higher HbA1c in older Indians may reflect better nutritional status, complicating the relationship [4, 7]. This paradox likely reflects the complex epidemiological transition underway in India, where obesity and metabolic syndrome co-exist with undernutrition, particularly in rural settings.

Educational attainment emerged as the strongest modifiable predictor in our study (aOR 3.87 for no formal education vs. ≥10 years). This is consistent with the cognitive reserve hypothesis: education builds neuronal redundancy that delays clinical expression of dementia pathology. The extraordinary educational disadvantage of older rural women in India—many of whom had no formal schooling—likely explains a significant portion of the gender gap observed in our data (aOR 2.18 for female sex). Interventions targeting mid-life educational attainment and lifelong learning may therefore serve a dual purpose in closing gender and rural–urban cognitive health disparities.

Depression was a strong independent predictor of cognitive impairment (aOR 1.83), consistent with extensive longitudinal evidence on bidirectional relationships between late-life depression and dementia. Given the high prevalence of depressive symptoms observed in rural participants (29.9%), and the limited availability of mental health services in rural India, integrated geriatric–mental health screening at the Ayushman Bharat Health and Wellness Centres represents a feasible policy response.

This study has several limitations. First, the cross-sectional design precludes causal inference; temporality between cardiometabolic exposures and cognitive outcomes cannot be established. Longitudinal follow-up using LASI Wave 2 data (2022–24) will be essential to disentangle directionality. Second, the cognitive instrument, though adapted from the HRS battery, relies on a composite index that does not distinguish between dementia subtypes (Alzheimer’s disease, vascular dementia, Lewy body dementia). Third, cardiometabolic exposures were self-reported, introducing recall and ascertainment bias, particularly for hypertension and diabetes. Future analyses incorporating LASI biomarker data (blood pressure, fasting glucose, CRP, haemoglobin) will strengthen causal interpretation. Fourth, state-level heterogeneity within India is substantial, and Tamil Nadu—with relatively higher health literacy—may differ from national estimates.

## 5. CONCLUSIONS

Cognitive impairment in rural India is predominantly driven by a convergence of low educational attainment, female sex disadvantage, hypertension, tobacco use, physical inactivity, and depression. These are, with the exception of sex and age, modifiable risk factors accessible to primary health care intervention. The integration of geriatric cognitive screening within existing National Programme for Non-Communicable Diseases (NP-NCD) platforms, coupled with community-based cardiometabolic risk reduction at Health and Wellness Centres, offers a pragmatic, scalable strategy to reduce the rural dementia burden. Longitudinal analyses using LASI Wave 2 are needed to confirm directionality and quantify attributable fractions for policy prioritisation.

## Supplementary Files

This is a list of supplementary files associated with this preprint. Click to download.

- BMCLASISTUDYSUPPLEMENTARYFILE.docx

## Figures and Tables

**Table 1:** Baseline characteristics of the study population stratified by rural–urban residence

Characteristic	Overall (N = 31,464)	Rural (n = 22,108)	Urban (n = 9,356)	p-value
Age (years), mean ± SD	68.4 ± 7.2	68.7 ± 7.4	67.7 ± 6.8	< 0.001
Female, n (%)	16,366 (52.0)	11,571 (52.3)	4,795 (51.2)	0.102
Education (years), mean ± SD	4.1 ±5.1	3.0 ± 4.6	6.8 ± 5.5	< 0.001
Hypertension, n (%)	11,847 (37.7)	7,761 (35.1)	4,086 (43.7)	< 0.001
Diabetes mellitus, n (%)	5,148 (16.4)	2,873 (13.0)	2,275 (24.3)	< 0.001
Current tobacco use, n (%)	9,439 (30.0)	7,266 (32.9)	2,173 (23.2)	< 0.001
Physical inactivity, n (%)	13,830 (44.0)	8,844 (40.0)	4,986 (53.3)	< 0.001
Depression (GDS ≥ 3), n (%)	8,814 (28.0)	6,612 (29.9)	2,202 (23.5)	< 0.001
Cognitive impairment, n (%)	3,385 (10.8)	2,820 (12.8)	565 (6.0)	< 0.001
Composite cognitive score, mean ± SD	25.4 ± 8.1	23.9 ± 8.0	29.1 ± 7.6	< 0.001

SD: standard deviation; GDS: Geriatric Depression Scale. p-values from chi-squared tests (categorical) and independent t-tests (continuous).

**Table 2:** Logistic regression analysis of determinants of cognitive impairment (N = 31,464)

Variable	Crude OR (95% CI)	Adjusted OR (95% CI)	p-value
***SOCIODEMOGRAPHIC***			
Age (per 5-year increment)	1.48 (1.42–1.54)	1.31 (1.26–1.37)	<0.001
Female sex	2.63 (2.43–2.84)	2.18 (1.99–2.38)	<0.001
No formal education (Ref: ≥10 years)	4.92 (4.29–5.65)	3.87 (3.35–4.47)	<0.001
Rural residence (Ref: urban)	2.27 (2.07–2.49)	1.68 (1.52–1.87)	<0.001
**CARDIOMETABOLIC RISK FACTORS**			
Hypertension	1.23 (1.14–1.33)	1.19 (1.10–1.29)	<0.001
Diabetes mellitus	1.08 (0.97–1.20)	1.11 (0.99–1.24)	0.072
Current tobacco use	1.31 (1.21–1.42)	1.26 (1.16–1.37)	<0.001
Physical inactivity	1.44 (1.33–1.56)	1.28 (1.18–1.38)	<0.001
**BEHAVIOURAL/PSYCHOSOCIAL**			
Depression (GDS ≥ 3)	2.11 (1.95–2.28)	1.83 (1.68–1.99)	<0.001
Social isolation	1.57 (1.44–1.72)	1.38 (1.26–1.52)	<0.001

OR: Odds Ratio; CI: Confidence Interval. Reference categories: male sex, urban residence, ≥ 10 years education, no hypertension, no diabetes, no tobacco use, physically active, GDS < 3. Adjusted model controls for all variables listed.

## Data Availability

The LASI Wave 1 dataset is publicly available upon registration at the International Institute for Population Sciences (IIPS), Mumbai (https://www.iipsindia.ac.in/lasi). Analytic code is available from the corresponding author on reasonable request.
